# Neural Processing of Repeated Search Targets Depends Upon the Stimuli: Real World Stimuli Engage Semantic Processing and Recognition Memory

**DOI:** 10.3389/fnhum.2018.00460

**Published:** 2018-11-15

**Authors:** Trafton Drew, Lauren H. Williams, Christopher Michael Jones, Roy Luria

**Affiliations:** ^1^Psychology Department, University of Utah, Salt Lake City, UT, United States; ^2^Department of Psychology, The Ohio State University, Columbus, OH, United States; ^3^Sagol School of Neuroscience and the School of Psychological Science, Tel Aviv University, Tel Aviv, Israel

**Keywords:** visual working memory, visual search, ERPs, CDA, N2pc, anterior N2, N400, LPC

## Abstract

Recent evidence has suggested that visual working memory (VWM) plays an important role in representing the target prior to initiating a visual search. The more familiar we are with the search target, the more refined the representation of the target (or “target template”) becomes. This sharpening of the target template is thought to underlie the reduced response time (RT) and increased accuracy associated with repeatedly searching for the same target. Perhaps target representations transition from limited-capacity VWM to Long-Term Memory (LTM) as targets repeat. In prior work, amplitude of an event-related potential (ERP) component associated with VWM representation decreased with target repetition, broadly supporting this notion. However, previous research has focused on artificial stimuli (Landolt Cs) that are far removed from search targets in the real world. The current study extends this work by directly comparing target representations for artificial stimuli and common object images. We found VWM representation follows the same pattern for real and artificial stimuli. However, the initial selection of the real world objects follows a much different pattern than more typical artificial stimuli. Further, the morphology of nonlateralized waveforms was substantially different for the two stimulus categories. This suggests that the two types of stimuli were processed in fundamentally different ways. We conclude that object type strongly influences how we deploy attentional and mnemonic resources prior to search. Early attentional selection of familiar objects may facilitate additional LTM processes that lead to behavioral benefits not seen with more simplistic stimuli.

## Introduction

We conduct hundreds of visual searches every day, from the mundane (milk in the refrigerator) to the vitally important (lesions in a medical image). What role does memory play during this important activity? This question has been the topic of a great deal of research in recent years. When considering visual search, it is important to delineate distinct phases of the task that may tap into different aspects of cognition. Unsurprisingly, most of the research has focused on the “search” phase of visual search. During this phase, there is controversy with respect to the role that memory plays. Despite claims that “visual search has no memory” (Horowitz and Wolfe, 1998), the current consensus is that although memory for what areas have already been searched is quite limited, memory does appear to play a role in helping searchers decide at what point they can stop searching (Peterson et al., 2001; Kok et al., 2017).

Although there is controversy with respect to the role that visual working memory (VWM) plays during the act of searching for a target, there is a great deal of converging evidence that VWM plays an important role in preparing to search for a specific target. During this time, the subject knows the target but cannot yet begin searching. This phase of visual search has been the focus of foundational work in the working memory literature (Chelazzi et al., 1993, 2001; Chelazzi et al., 1998). In one example of this work, Chelazzi and colleagues trained nonhuman primates to respond to a specific target after a blank delay interval. The target for each trial was cued prior to the delay interval. During this interval, researchers typically find what is referred to as “delay activity” specific to target representation. This activity is thought to be the neural signature of ongoing working memory maintenance of the target representation in the absence of visual stimulation (Chelazzi et al., 1993). Early evidence for these effects was primarily in inferotemporal cortex, but more recent examinations have suggested that delay activity is widely distributed throughout the cortex (Dotson et al., 2018).

The delay activity observed in these unit recording studies is the namesake of an electrophysiological correlate of VWM in humans, the CDA for Contralateral Delay Activity. This component was discovered using a change detection task where subjects are asked to encode a variable number of targets, hold them in VWM during a delay interval, and then report if any of the targets have changed on a test screen. The amplitude of CDA increases with memory set size and reaches asymptote when an individual’s VWM capacity has been reached (Vogel and Machizawa, 2004; McCollough et al., 2007; Drew and Vogel, 2008).

Recently, this component was employed to explore how the role of VWM changes when the search target is repeated. Carlisle et al. (2011) found that CDA amplitude decreased with target repetition, suggesting that the role of VWM decreases as well. In this study, subjects were shown a target (a Landolt C of a specific orientation). After a delay, they had to search an array of Cs for the target C (present on 50% of trials). The authors argue that as the target repeats and becomes more familiar, it is no longer necessary to actively represent target information in VWM. Moreover, they found that a neural index of Long-Term Memory (LTM), the P170 (Voss et al., 2010) increased with target repetition, suggesting that as VWM representation decreases, LTM representation increases (Carlisle et al., 2011; Reinhart and Woodman, 2013; Woodman et al., 2013). This transition from VWM to LTM representation may represent a pathway for information to be consolidated into a durable format for learning.

In the current study, we aimed to extend this work by examining how this pattern of data changes when the materials employed more closely resemble the search targets we experience in the real world. As Gunseli et al. (2014) previously highlighted, the initial demonstrations of this effect relied upon pop-out search arrays where the target was a single colored item amidst black items. Therefore, while the cue was necessary to determine the target had the correct orientation, it was not necessary to find the target location. It was therefore unclear whether the observed decrease in CDA amplitude was due to preparation for a target identification task, search, or some combination of the two. Gunseli et al. (2014) addressed this concern by comparing the electrophysiological response when the search array contained either a distinct (replicating prior work) or nondistinct target. The nondistinct target created a more difficult search where Response Time (RT) increased when more items were on the screen. Despite large behavioral differences which suggested that “search” played a larger role in the nondistinct condition, CDA amplitude was unaffected by this manipulation: there was significant decrease in CDA amplitude for both conditions as the target repeated. However, these authors observed a significant decrease in the P170 with target repetition when there were 8 potential targets, but not when there were 4 potential target Cs. One possible explanation for this finding is that when there are few (4) potential targets, the subject can consolidate each of these potential targets over the course of the experiment, resulting in no reliable effect for target repetition. This suggests that the P170 effect may be more pronounced when there are more potential targets.

While the CDA and P170 are thought to represent VWM representation and the accumulation of information in LTM, respectively, this paradigm also allows researchers to examine the attentional deployment to the onset of the target by examining the N2pc. This is a negative-going contralateral potential that has a similar (though not identical) distribution (lateral occipital electrodes) to the CDA (McCollough et al., 2007). This component is thought to represent focusing of visual attention within a complex visual scene. Traditionally, experimenters analyzed this component by time-locking to the onset of a search array and N2pc amplitude is taken to denote an electrophysiological correlate of deploying attention to the target item. More recently, researchers have found that the amplitude of this component scales with the number of items being attended in a subitizing task (Ester et al., 2012), and is larger when the target is more discriminable from distractors (Zhao et al., 2011). Prior work on target repetition with artificial stimuli found no effect of repetition on N2pc amplitude (Carlisle et al., 2011; Woodman et al., 2013; Gunseli et al., 2014). However, we wondered whether this pattern of results may depend upon the use of artificial stimuli. There is some evidence that the N2pc is sensitive to changes in LTM representation, which may lead to increased N2pc amplitude (Patai et al., 2012). However, this evidence comes from detection of a target in a search array, rather than the initial encoding of the target. From this work, it is clear that LTM can enhance attentional guidance, leading to a larger N2pc and higher d’, but it is currently unclear how LTM affects the initial encoding of a repeated target that is a real-world item.

Although most of the prior research has used artificial Landolt C stimuli that result in a limited number (4 or 8) of possible targets, Reinhart and Woodman (2014) extended this work to real world objects in an experiment that explored the effect of transcranial direct-current stimulation. For current purposes, we will focus on the “sham” condition, where there was no transcranial stimulation. The target in this experiment was a one of 8 potential images from a “dog” or “cat” category that was cued with a red or green circle. After a delay, the search array appeared with 10 inanimate objects in black circles, one red circle, and one green circle with an item from the “dog” or “cat” category. The subject had to determine if the item in the cued color matched the target or did not. Importantly, this design creates a “pop-out” search where the subject would likely be unaffected by the number of distractors in the black circles. The task is thus very similar to a delayed-match to sample task used for some of the initial demonstrations of the delay-activity (Chelazzi et al., 1993). In addition, by restricting the number potential targets to 8, the authors matched prior work with artificial stimuli, but this leaves open the possibility that we may see a dramatically different response when this restriction is lifted. The results were broadly similar to prior work with artificial stimuli: CDA amplitude decreased with repetition and P170 amplitude increased. Interestingly, the N2pc appears to increase with repetition, but it unclear whether this effect is statistically significant because the authors were focused on how the pattern of repetition effects changed with tDCS stimulation. Similarly, the authors did not compare the electrophysiological response across experiments where the stimuli were either artificial (their Experiment 1) or real world images (Experiment 2).

### Current Study and Predictions

The current study aimed to more closely examine the differences elicited by artificial and real world stimuli. In contrast to the prior work using real world stimuli, we did not restrict the number of potential targets, meaning that the subject was unlikely to encounter a given target for more than one epoch of time. For both artificial and real world stimuli, we did not cue the potential target in the search array, meaning that subjects needed to covertly search the array for the presence of the target before responding. This feature of the procedure was designed to ensure that the subject needed to encode a high-resolution representation of the target in order to complete the task.

Landolt Cs share common features and lack meaningful semantic content. In contrast, the real world objects we employed share few features and are associated with unique semantic content. How will these differences manifest in the resultant behavior and electrophysiological response? Given the high degree of feature overlap with Landolt Cs and low degree with real world objects, we predicted that the task would be more difficult (longer RT and lower accuracy) with artificial stimuli. We hypothesized that the semantic content and unique features associated with real world object targets would lead to stronger engagement with LTM processing. Thus, we predicted real world objects would yield a larger modulation of the P170 component thought to denote LTM encoding. We also predicted that the additional LTM processing would enable VWM resources to be offloaded more quickly with real world objects, resulting in a steeper decrease in CDA amplitude with target repetition. Along similar lines, we predicted that amplitude of the Late Positive Component (LPC), which is sensitive to explicit memory of previously encountered items (Rugg and Curran, 2007), would be sensitive to target repetition. Specifically, as prior research has suggested that the LPC is an index of both VWM representation and anticipated difficulty (Gunseli et al., 2014), we predicted that LPC amplitude would decrease with target repetition and be generally more positive for Landolt Cs.

## Materials and Methods

### Experiment 1

#### Participants

In Experiment 1, there were 23 total participants. Participants were recruited through fliers or the University of Utah undergraduate participant pool. Participants were compensated with course credit or paid $10–15 per hour. The study was approved by the University of Utah’s Institutional Review Board and all participants provided informed consent. Fifteen of these participants were used in data analysis (mean age = 24, age range 18–35, 5 female, 10 male). All participants were neurologically normal and not colorblind. Participants were excluded from analysis if more than 30% of trials were flagged during artifact detection. Six participants were excluded because of excessive blinks and eye-movements, and two participants were excluded because they did not complete the experiment. Prior work examining the effect of target repetition on CDA amplitude with two stimulus conditions examined 12 participants in Experiment 1 and 18 in Experiment 2. Based on the observed effect size (η*_p_*^2^= 0.41 and 0.29, respectively) of the Repetition by CDA amplitude effect we aimed for 16 good participants in both of our experiments.

#### Procedure

**FIGURE 1:**
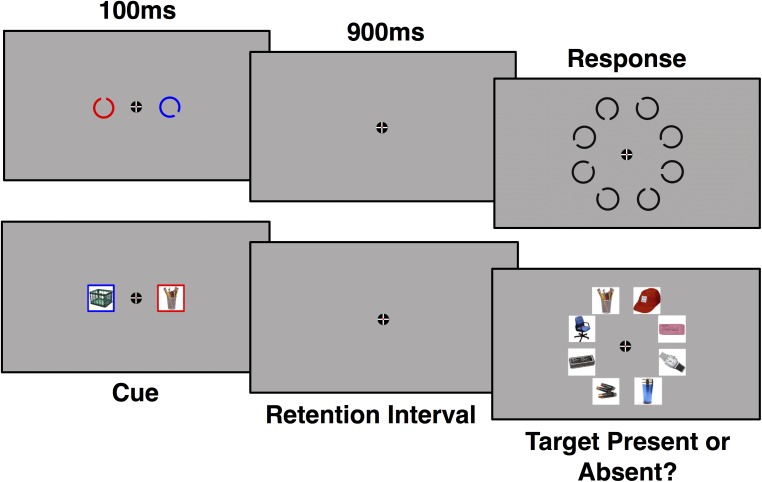
Example of a trial using Landolt C (top) and real world object (bottom) stimuli. ERP waveforms were time-locked to onset of the cue for a period of 1000 ms. Targets were present on half of trials.

All experimental tasks and stimuli were presented in Psychtoolbox using MATLAB (Brainard, 1997; Pelli, 1997; Kleiner et al., 2007) on a 24^′′^ ASUS VG248QE 144 Hz monitor. Participants sat approximately 36^′′^ away from the monitor. Experiment 1 utilized two types of stimuli: Landolt Cs and images of real world objects (Brady et al., 2008). There was 1 short practice block prior to the experimental trials which was not recorded for analysis. There were 14 blocks of 48 trials for each stimulus type, for a total of 1344 trials, 112 trials for each repetition for both stimulus types. Stimulus order was counterbalanced across participants. At the beginning of the experiment, participants were instructed to attend to either the red cue or the blue cue (see Figure 1). Color was counter-balanced across participant. Participants were instructed to fixate on a cross for the duration of each trial. Each trial was preceded by a randomly jittered (1200–1600 ms) inter-trial interval where only the fixation cross was visible. The target was then cued for 100 ms followed by a 900 ms retention interval. Cued items were randomly presented on opposite sides of the screen (∼1.9° of visual angle from the fixation point). Landolt Cs were cued by object color, while the images of real-world objects were cued by a colored frame around the object. Both Landolt Cs and the images subtended ∼2.8° of visual angle. After the retention interval, a search array of 8 objects (each object in the array subtended ∼3.5° of visual angle) was presented until the participant made a response. Participants were instructed to press “j” if the target was present and “f” if the target was absent, which was randomly determined on each trial. Targets were present on 50% of all trials and could occur anywhere in the search array (rather than being lateralized to the attended side). Each target object was repeated for six consecutive trials before a new target was presented. Participants were notified of the target change during a jittered (1200–1600 ms) inter-trial interval, during which the participant was allowed to blink, via the word “change” displayed under the fixation cross before the new target was cued.

### Experiment 2

#### Participants

In Experiment 2, there was a total of 20 participants. The study was approved by the University of Utah’s Institutional Review Board and all participants provided informed consent. Four participants were excluded from data analysis due to excessive blinks and eye movements. Data from sixteen participants (mean age = 25, age range 18–36, 7 female/9 male) were used for analysis. One participant took part in Experiments 1 and 2.

#### Procedure

The procedure for Experiment 2 was the same as Experiment 1 with the exception of the following: participants completed 16 blocks with 48 trials, for a total of 768 trials, 128 trials for each target repetition. Participants only searched for the real world object stimuli (Brady et al., 2008), and the search array contained 12 objects. Both the target cue and the objects in the search array subtended ∼3.1° of visual angle.

### Electrophysiological Analysis

#### Electrophysiological Recording

The EEG was recorded from 32 Ag/Cl active scalp electrode sites using the International 10/20 system (Brain Products’ actiCAP). The EEG was amplified with Brain Products’ ActiCHamp system and digitized at 500 Hz. Conductive gel and light scalp abrasion were used to reduce impedance below 15 kOhms. Electrodes were referenced online to the average of the left and right mastoids. The EEG data were processed offline using EEGLAB and ERPLAB in MATLAB (Delorme and Makeig, 2004; Lopez-Calderon and Luck, 2014).

#### Filtering and Artifact Rejection

Epochs with blinks, eye-movements, excessive noise, or other large artifacts were excluded from the analysis. HEOG was measured using two active electrodes placed 1cm lateral to external canthi. Blinks were detected using frontal electrodes (FP1/2) above the eyes. A moving window was used to detect blinks and other artifacts on our critical electrodes (P7/8, PO7/8). In addition, a step function was used on the HEOG channels to detect eye movements. The standard thresholds for artifact detection were 140 μV for the moving window and 40 μV for eye movements. However, these thresholds were adjusted for individual participants as necessary to increase the signal to noise ratio. On average, this led to a rejection rate of 14% of trials in Experiment 1 and 15% in Experiment 2. A Butterworth high pass filter with a half-amplitude cutoff of 0.01 Hz was applied to the continuous EEG data. A 30 Hz low pass Butterworth filter was applied to the epoched data for plotting purposes only. Statistical analyses were performed on the unfiltered ERP data.

#### ERP Analyses

ERP waveforms were time-locked to the onset of the target cue and baselined to the 200 ms before the onset of the target cue. We calculated lateralized components (N2pc and CDA) by averaging the data from PO7/8 and P7/8 and subtracting the contralateral electrode sites from the ipsilateral electrode sites with respect to cued target location. These electrodes were selected because they elicited the largest CDA and N2pc response irrespective of repetition or stimulus set. As in Luck and Hillyard (1994), mean amplitude for the N2pc was measured from 200 to 300 ms after the onset of the target cue. Following Vogel and Machizawa (2004), mean amplitude for the CDA was measured from 300 to 1000 ms after the onset of the target cue. Following prior research (Gunseli et al., 2014), we measured nonlateralized components (P170, P3b, and LPC) at electrode sites Cz, Pz, and Fz. We used a 150–200 ms window for the P170, a 300–400 ms window for the P3b, and a 475–700 ms window for the LPC (Gunseli et al., 2014). Based on prior research (e.g., Folstein, 2008) and visual inspection, we examined the N2 during a 200–250 ms time window at the Cz electrode.

## Results and Discussion

For both experiments, we applied a Greenhouse-Geisser correction to all repeated measures ANOVAs where the sphericity assumption was violated.

### Experiment 1: Behavior

**FIGURE 2:**
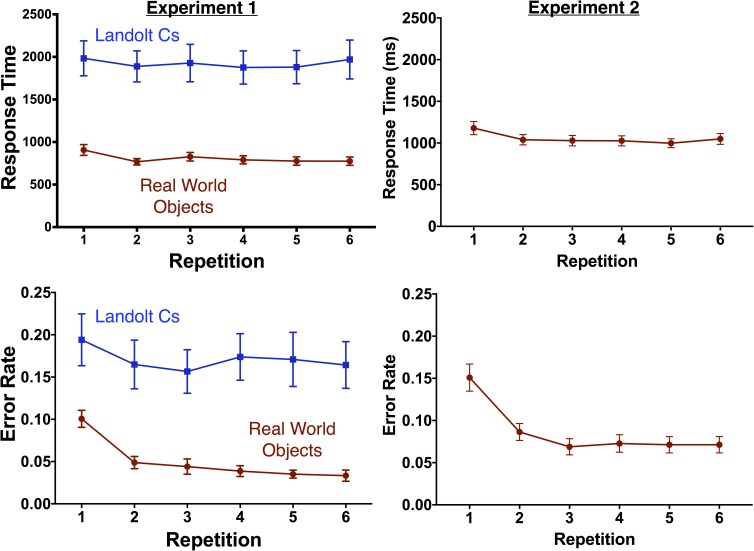
Behavioral results by repetition for Experiment 1 (left) and Experiment 2 (right). Error bars indicate the standard error of the mean.

To analyze behavior, we computed a repeated measures ANOVA with repetition and stimulus type as factors. As expected, the Landolt C stimuli were associated with slower [*F*(1, 14) = 43.3, *p* < 0.001] and less accurate [*F*(1, 14) = 25.1, *p* < 0.001] responses (see Figure 2). There was a significant effect of target repetition on both RT [*F*(2.7, 38.5) = 3.5, *p* = 0.027] and error rate [*F*(2.9, 41.1) = 11.5, *p* < 0.001]. There was a significant interaction between target repetition and stimulus type for error rate [*F*(3.6, 50.2) = 3.8, *p* = 0.012] but not RT [*F*(2.9, 40.1) = 0.93, *p* = 0.43]. In the object condition, there was a significant effect of repetition on accuracy [*F*(3.3, 46.0) = 26.67, *p* < 0.001] and performance was worse for the first instance of the target than any of the subsequent repetitions (Dunnett’s multiple comparison test, all *p*s < 0.001). In contrast, there was no effect of repetition on accuracy in the Landolt C condition [*F*(4.0, 55.3) = 2.3, *p* = 0.07] and performance on the first instance of a target was equivalent to all repetitions except the third repetition (*p* = 0.029).

### Experiment 1: Electrophysiology

#### Contralateral Effects

**FIGURE 3:**
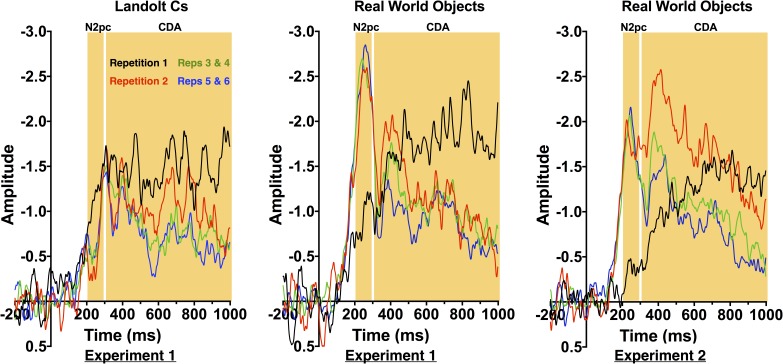
Contralateral-Ipsilateral difference waves by repetition for Experiments 1 and 2.

Figure 3 shows ERP waveforms averaged across electrode pairs P7/8 and PO7/O8. Following prior work, for statistical analyses, we compared Repetition 1, 2 and the average of 3 and 4 and 5 and 6 (Gunseli et al., 2014). Replicating prior work, CDA amplitude decreased with repetition [*F*(3.2, 44.3) = 3.6, *p* = 0.018]. However, there was no effect of stimulus type [*F*(1, 14) = 1.6, *p* = 0.232] and no interaction between the two factors. This suggests that the rate of decrease was equivalent for both stimulus types. This therefore suggests that transfer from VWM to a more durable representation is equivalent across the two stimulus types.

**FIGURE 4:**
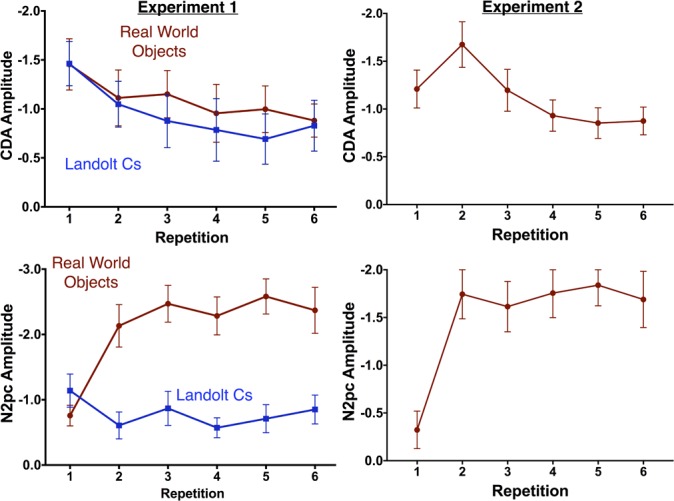
Mean amplitude of the CDA (top) and N2pc (bottom) by repetition for Experiments 1 (left) and 2 (right). Error bars indicate the standard error of the mean.

In contrast, the N2pc exhibited a strikingly different pattern of results for the two stimulus conditions. Overall, there was a significant effect of repetition [*F*(3.8, 53.1) = 3.9, *p* = 0.009] and stimulus [*F*(1, 14) = 40.253, *p* < 0.001], and a significant interaction between the two factors [*F*(4.5, 63.6) = 13.9, *p* < 0.001]. As can be observed in Figures 3, 4, N2pc amplitude is generally higher for the objects than the Landolt Cs. Moreover, the interaction appears to be driven by changes in the object condition [*F*(3.8, 53.7) = 13.23, *p* < 0.001] while amplitude in the Landolt C condition did not change with repetition [*F*(3.7, 51.7) = 1.53, *p* = 0.21].

The lack of an effect of repetition on N2pc amplitude is consistent with prior work using artificial stimuli (Carlisle et al., 2011; Gunseli et al., 2014). The clear modulation of the N2pc amplitude with real world stimuli suggests that the initial deployment of attention is fundamentally different with these more realistic stimuli. One possibility is that this modulation denotes a level of recognition for repeated targets that does not take place for more artificial, less unique stimuli. From this interpretation, the N2pc might serve as a signature of LTM engagement that occurs when a repeated target is recognized as having been recently processed.

#### Nonlateralized Effects

**FIGURE 5:**
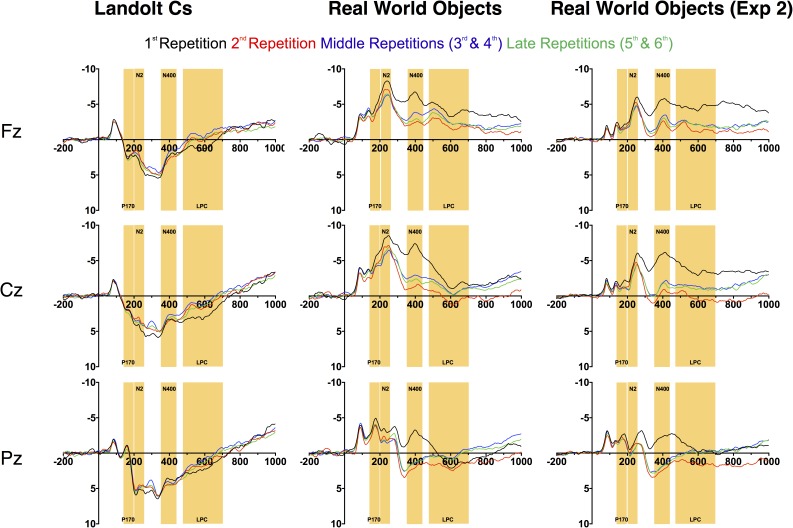
Non-lateralized waveforms for Experiments 1 and 2.

Nonlateralized waveforms for Fz, Cz, and Pz electrodes can be seen in Figure 5. Although the lateralized waveforms are broadly similar across stimulus type (though with important differences highlighted above), it is immediately clear that stimulus type had a very large effect on the nonlateralized waveforms. The N1 response, which is associated with the initial deployment of attention, was significantly more negative for Objects than Landolt Cs [*F*(1, 14) = 15.37, *p* = 0.002], but the effect was unaffected by repetition [*F*(3.9, 55.2) = 1.14, *p* = 0.35] and the two factors did not interact [*F*(3.2, 45.3) = 0.85, *p* = 0.49]. This effect is likely driven by low-level differences in the two types of stimuli: the objects are more complex, colorful and contain larger variation in contrast.

After the N1, there are large morphological differences that continue throughout our time window. Previous work in this paradigm has observed a P170. We replicate this effect with Landolt Cs. However, real world objects appear to elicit a large N170 that can be observed over medial-frontal electrodes (see Figure 5; Bentin et al., 1996; Bentin and Deouell, 2000). Later, the positive amplitude p3b component (300–400 ms) that we and others (Gunseli et al., 2014) observe in the Landolt C condition is also inverted: we observe a negative component during this time period in the real world object condition. We suspect that these morphological differences are due to the large differences in semantic content across the two conditions. There is a very little semantic content associated with a single Landolt C. Real world objects are associated with a wealth of semantic information which may be driving these large differences. We return this issue in the discussion.

**FIGURE 6:**
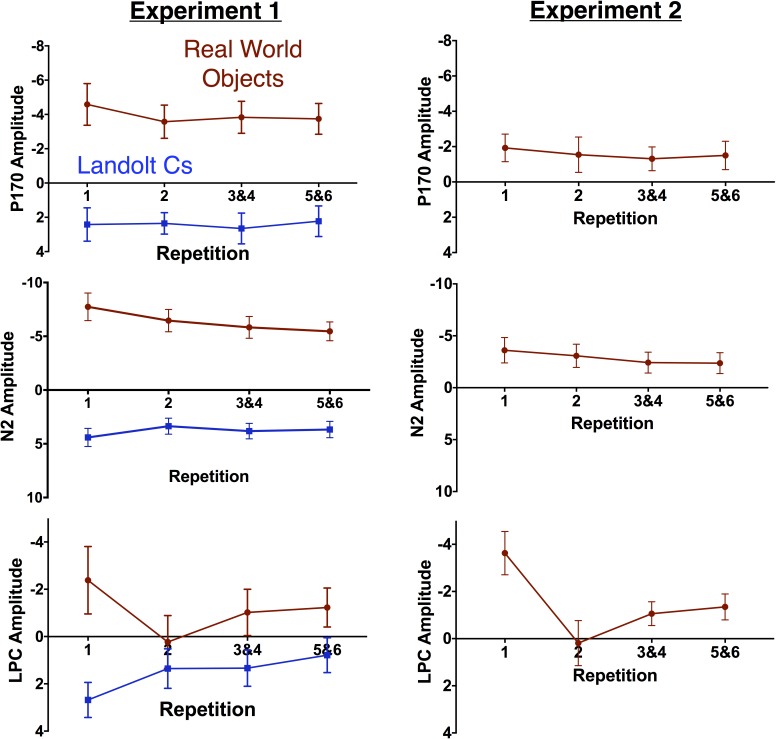
Mean amplitude of the non-lateralized components by repetition for Experiments 1 (left) and 2 (right). Error bars represent the standard error of the mean.

Prior work has suggested that the effect of repetition may be influenced by the number of potential targets. Gunseli and colleagues found that amplitude of the P170 was unaffected by repetition when there were 4 potential Landolt C targets. However, there was a significant effect when there were 8 potential Landolt C targets. We therefore hypothesized that the P170 effects would be larger for targets that were objects that did not repeat throughout the experiment than Landolt Cs with 6 potential orientations. As prior work found that the effect was maximal at Fz, we focused on this electrode. There was a large effect of stimulus [*F*(14, 1) = 38.9, *p* < 0.001], but no effect of repetition [*F*(3, 42) = 1.3, *p* = 0.29] and no interaction [*F*(3, 42) = 1.2, *p* = 0.33, see Figure 6]. Thus, we did not find evidence in favor of our hypothesis that more potential targets would lead to a larger effect of repetition on the putative index of LTM consolidation, the P170. More generally, we did not replicate the previously observed effect of repetition on P170 amplitude.

Immediately after the time-window (150–200 ms) typically associated with the P170, we observe a large anterior N2 in the Object, but not Landolt C condition. We focused on the Cz electrode, where this effect appeared to be maximal. Here there was an effect of stimulus type [*F*(1, 14) = 77.6, *p* < 0.001] and repetition [*F*(3, 42) = 3.4, *p* = 0.03] and the two factors interacted significantly [*F*(3, 42) = 8.1, *p* = 0.002]. The repetition effect appears to be largely driven by the first instance of the object, which elicits a larger N2 deflection than any of the other conditions (Dunnett’s multiple comparison test, all *p*s < 0.05). We believe this is consistent with data that has suggested that the anterior N2 is sensitive to novelty (Folstein and Van Petten, 2007). After 6 repetitions with the same object serving as the target, the appearance of a new object appears to trigger this novelty response. It appears that the appearance of a new Landolt C target item is not sufficiently novel to trigger this response. This is consistent with early work from Courchesne and colleagues (Courchesne et al., 1975), who found that amplitude of anterior N2 amplitude was higher for a complex novel stimulus (complex randomly colored patterns) than for equally uncommon simple (black and white shapes and words) stimuli.

Next, we examined the amplitude of the LPC. Gunseli and colleagues previously found that this component was sensitive to both target repetition and task difficulty (Gunseli et al., 2014). They argue that the amplitude of this component is thus sensitive to both working memory maintenance and anticipated effort. In our experiment, Landolt Cs were associated with longer RT and lower accuracy. As a result, we predicted that the LPC would be more positive for Cs than real world objects. We also predicted that LPC amplitude would decrease with repetition for both types of stimuli. Our predictions were partially confirmed by the data. Overall, there was a significant effect of stimulus type [*F*(1, 14) = 11.7, *p* < 0.001], but not repetition [*F*(3, 42) = 1.54, *p* = 0.22] and the two factors interacted significantly [*F*(3, 42) = 9.8, *p* < 0.001, Figure 6]. Consistent with our predictions, real world objects were less positive than Cs. Amplitude appears to be sensitive to repetition for both stimulus types, but in opposite directions. Consistent with previous results, LPC amplitude decreases with repetition with Landolt Cs [*F*(3, 42) = 7.2, *p* < 0.001]. On the other hand, amplitude *increases* with repetition for real world objects [*F*(3, 42) = 4.0, *p* = 0.014]. A canonical interpretation of LPC would argue that this suggests that effort increases with target repetition for real world objects, but this discounts the large morphological differences observed for the two types of stimuli. Moreover, it is at odds with the behavioral data: both accuracy and RT indicate that the Landolt Cs was more difficult. In fact, it may not be appropriate to refer to amplitude during this time period as LPC for objects given that amplitude is generally negative, rather than positive.

**FIGURE 7:**
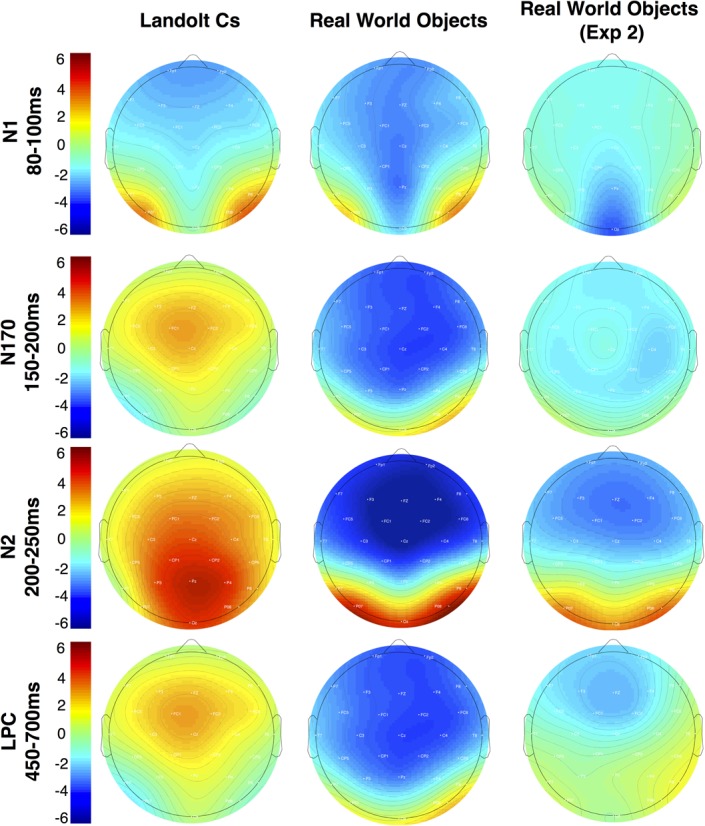
Topographic maps of mean amplitude for the non-lateralized components for Experiments 1 and 2.

Perhaps a better description of this activity is a negative slow wave (NSW). Prior work has found that amplitude of this component, which is measured during the delay interval of a variety of delay match to sample tasks, increases with the amount of information to be held in VWM (Ruchkin et al., 1997). Scalp topography of this component appears to vary with the stimulus materials. With objects, prior work has found that the wave is focused on mid-frontal electrodes (Mecklinger and Pfeifer, 1996). This is consistent with the activity associated with real world objects observed here (see Figure 7). The fact that we appear to be observing LPC activity that *decreases* with target repetition and/or effort with Landolt Cs and NSW activity that *increases* with real world objects in the same subject using a matching task again highlights the large differences in processing that emerge when highly controlled laboratory stimuli are replaced with meaningful real world objects.

**FIGURE 8:**
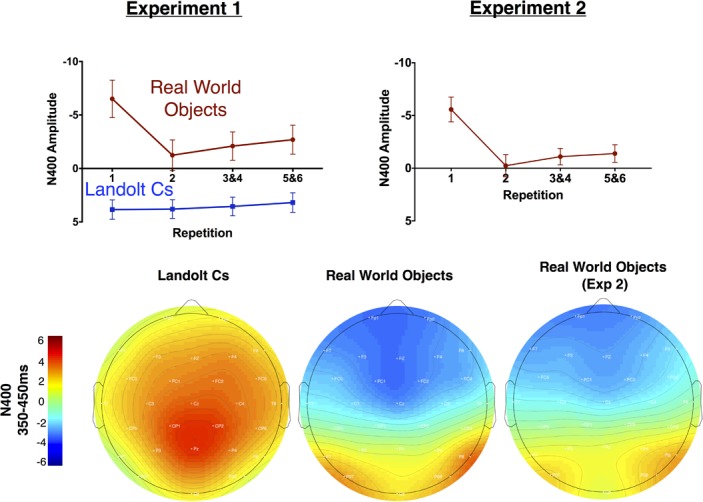
Mean amplitude of the N400 by repetition for Experiments 1 and 2 (top). Topographic maps of mean amplitude for the N400 for Experiments 1 and 2 (bottom).

Perhaps most strikingly, we observed what appears to be a large N400 that is sensitive to repetition in the Real World Objects condition, but the waveform is positive and not sensitive to repetition in the Landolt C condition (see Figures 5, Figures 8). Overall, we found that during the 350–450 ms time window, there was a significant effect of repetition [*F*(3, 42) = 10.6, *p* < 0.001] and stimulus [*F*(1, 14) = 43.9, *p* < 0.001] and the two factors interacted significantly [*F*(3, 42) = 17.9, *p* < 0.001]. When we focus on the Landolt C condition, there was no effect of repetition during this window [*F*(3, 42) = 1.0, *p* = 0.40]. When we focus on the Real World Objects, we found an effect that similar to the anterior N2: there was significant effect of repetition [*F*(3, 42) = 17.3, *p* < 0.001] and the first instance of the target evoked was more negative than any of the subsequent repetitions (all *p*s < 0.001). Although the N400 was first characterized in response to words, more recent work has shown that it is a general electrophysiological response to essentially all meaningful stimuli, regardless of modality (Kutas and Federmeier, 2011). The N400 is thought to index relatively automatic semantic processing of currently relevant stimulus categories. In memory research, the repeated memoranda are associated with reduced (more positive) N400 amplitude. One theory for the cause of these effects is that the N400 reduction is associated with greater fluency of semantic processing for previously processed stimuli, or *perceptual priming* (Kutas and Van Petten, 1990). From this perspective, the N400 is a useful index of familiarity (Voss and Federmeier, 2011). Thus, our data suggests that target repetition clearly modulates the subject’s familiarity with the target object, but only when the object in question is a Real World Object, rather than a Landolt C. We take this as further evidence that semantic processing is more deeply engaged when the targets are meaningful real world objects rather than tightly controlled laboratory stimuli.

### Summary

The primary motivation for Experiment 1 was to examine whether CDA and P170 amplitude would follow a different pattern in response to targets that were real world stimuli rather than simplistic laboratory stimuli. Although we found no evidence that this was the case, Experiment 1 yielded two unexpected results that we hoped to replicate in Experiment 2: the large increase in N2pc amplitude after the first presentation of a real world object target, and the large morphological differences in response to real world objects relative to the Landolt Cs in Experiment 1 and prior work from other labs using artificial stimuli in the task. The large morphological changes observed in response to our stimulus type manipulation led us to evaluate the Anterior N2 and the N400 and suggest that what was previously referred to as a the LPC might be better thought of as a NSW. These observations were largely based on visual inspection of the waveforms and topography of the effects. As such, they should be treated as exploratory. Experiment 2 allowed us to see whether these effects replicated with an independent sample of subjects.

### Experiment 2: Behavioral Results

Replicating Experiment 1, target repetition in Experiment 2 was associated with decreased RT [*F*(2.64, 39.6) = 13.1, *p* < 0.001] and fewer errors [*F*(2.4, 36.6) = 27.7, *p* < 0.001]. As in Experiment 1, performance was worse for the first instance of the target than any of the subsequent repetitions (Dunnett’s multiple comparison test, all *p*s < 0.001).

### Experiment 2: Electrophysiology

#### Contralateral Effects

Experiment 2 replicated both contralateral effects observed with real world objects in Experiment 1. One-way ANOVAs indicate that there was a significant effect of target repetition on CDA amplitude [*F*(3.4, 50.9) = 5.18, *p* = 0.002] and N2pc [*F*(2.8, 41.4) = 13.1, *p* < 0.001].

#### Nonlateralized Effects

As can be observed in Figure 5, the broad morphology for the real world objects in Experiment 1 and Experiment 2 is similar. This assessment is solidified by a pattern of qualitatively equivalent statistical effects. As in Experiment 1, there was no effect of target repetition on P170 amplitude [*F*(2.6, 39.1) = 1.1, *p* = 0.37]. N2 amplitude also showed a similar pattern: [*F*(1.9, 28.9) = 3.4, *p* = 0.05]. The LPC amplitude also followed a similar pattern to the real world objects in Experiment 1: repetition had a significant effect on LPC amplitude measured at the Cz electrode. Increased repetition led to a less negative amplitude during this time period [*F*(2.1, 31.0) = 13.1, *p* < 0.001]. As in Experiment 1, we believe that activity during this time window is better described as a NSW (Ruchkin et al., 1997), which decreases in amplitude as target repetition increases. Finally, as in Experiment 2, the N400 became less negative with repetition [*F*(1.9, 28.4) = 30.2, *p* < 0.001], and the first instance of a target was associated with a more negative response than any subsequent repetition (all *p*s < 0.001). In sum, Experiment 2 provides an independent replication of the unexpected results (modulation of the N2pc, N2, N400, and LPC with repetition) we observed in Experiment 1.

## General Discussion

When faced with a repeated target, electrophysiological evidence suggests that working memory representation of target information decreases, but performance improves. How could this be the case? Representation of the target must be off-loaded to alternative cognitive resources. Given VWM’s limited capacity, this process of offloading information from VWM to alternative representational space must be happening constantly, but this process is poorly understood (Rhodes and Cowan, 2018). In the current study, we examined how this process differs when using real world stimuli as compared to more simplistic laboratory stimuli that have been used in most prior research on this topic. We hypothesized that the additional semantic content and familiarity associated with real world objects would lead to a faster transition from VWM representation to LTM representation. Based on changes in CDA amplitude, this did not appear to be the case. In fact, CDA amplitude for the two conditions was remarkably similar given the large differences in stimulus attributes associated with the two stimulus types. Although these differences did not lead to any observable changes in the VWM representation, they did lead to an array of other unexpected effects that we replicated in Experiment 2:

(1) In contrast to prior work with artificial stimuli, we found that N2pc amplitude was sensitive to repetition, but only with real world objects. We found that the amplitude of the N2pc greatly increased after the first presentation of the target.

(2) We found a dissociation in the effect that target repetition had on the ability to correctly identify targets: Accuracy with real world objects increased with repetition, with most of the benefits concentrated between the first and second instance of the target.

(3) We observed large morphological differences in the activity elicited by Landolt Cs and Real World Stimuli. With objects, there was no evidence of previously reported P170 modulation and the waveform was generally more negative than the response we and others have observed in response to Landolt Cs.

### N2pc

There is a large literature devoted to using the N2pc to examine the neural underpinnings of search. The grand majority of this literature is confined to search arrays with simple stimuli and targets that either remain the same for the entire experiment or change on each trial. However, even those few papers that employ realistic objects tend to focus on the search array rather than preparatory activity prior to the search (Nako et al., 2014). One interpretation is that because they are more distinct, Real World Objects allow for better individuation and selection than Landolt Cs. Based on this interpretation, N2pc amplitude rises on the second instance of the target because it takes some experience with a complex target to individuate the features associated with the item. Another possibility is that the large increase in N2pc amplitude in response to previously observed targets is an early neural correlate of recognition: remembering searching for that particular red car on a previous trial. Importantly, this distinct signature is entirely absent with Landolt C stimuli, where the N2pc is unaffected by repetition and all targets were quite similar. Prior work examining the effect of target repetition (Carlisle et al., 2011; Reinhart and Woodman, 2013; Gunseli et al., 2014) may not have observed this effect because all of this prior work restricted the number of possible targets.

Our data suggest that an increase in N2pc amplitude to a repeated complex stimulus might be a signature of the first moment where LTM representations are brought to bear on the processing of a potential target. If this is the case, one would expect that this increase in N2pc amplitude should not depend upon repeating the target on consecutive trials. If the LTM memory trace is still accessible, N2pc amplitude should reflect this irrespective of whether the same target is repeated in a sequence of continuous trials or interspersed throughout an experiment. We are testing this hypothesis in ongoing work: osf.io/h5cfq.

### Behavior

In both experiments, we found a large accuracy increase from the first to second instance of real world objects. Accuracy was unaffected by repetition with the less distinct Landolt Cs. The unique benefit of searching repeatedly for a distinct real world object despite no observable differences in our measure of VWM engagement suggests that this benefit must be coming from a separate cognitive resource and perhaps the N2pc is an early signature of engagement of those resources. It is important to note that due to the high degree of similarity with distractors, the Landolt C task was significantly harder than the Real World Object task. It is therefore not the case that this effect is driven by there being more room for improvement with Real World Objects. Consistent with prior work, we observed a significant decrease in RT associated with repetition in the Landolt C condition. Thus, target repetition of both stimulus types was associated with some behavioral benefit, but the effect appears to be stronger when using Real World Objects. We speculate that the additional behavioral benefits associated with Real World Object repetition are due to recruitment of semantic processing associated with unique targets that is not possible when using simple laboratory stimuli. In fact, many researchers deliberately avoid using meaningful stimuli in order to avoid semantic associations, which may inflate capacity estimations (Luck and Vogel, 1997; Vogel et al., 2001). Although this approach arguably leads to more pure estimates of cognitive abilities, it also takes the resultant conclusions a step further away from the real world, where it is unusual to search for perfectly controlled stimuli that do not vary based on semantic content.

### Nonlateralized Activity

In light of the lack of differences in the modulation of the CDA across stimulus types, the morphological differences observed in nonlateralized activity are quite striking. The two waveforms appear to diverge completely after the early-evoked activity in the N1. At this point, Landolt Cs appear to elicit a P170 consistent with prior work (Woodman et al., 2013; Gunseli et al., 2014). Meanwhile, the Real World Object waveform shows no evidence of a P170. Instead, the waveforms stay negative during this time period and appear to proceed to a large anterior N2 modulation that is sensitive to target repetition. The Real World Object waveform stays mostly negative for the duration of our time window such that while previous work has examined an LPC during the later time window, activity during this time window appears to be more consistent with a NSW that has previously been associated with working memory representation and task difficulty (Ruchkin et al., 1997). These large differences may be best appreciated in the topographic maps in Figure 7.

We found an interesting dissociation between the response to repetition observed in the anterior N2 and N2pc. In both experiments, N2 response to objects was largest for the first target repetition and equivalent for all subsequent repetitions. In contrast, the N2pc response was *smallest* for the first instance of a new target in the object condition. This suggests that the anterior N2 is sensitive to the novelty of complex targets. In contrast, as outlined above, N2pc amplitude may represent an index of early recognition of a previously attended target with complex stimuli. If the anterior N2 is a valid measure of target novelty, the modulation of this component with real world objects, but not Landolt Cs, is consistent with the idea that the degree of semantic processing for these two types of stimuli is fundamentally different. This may, in turn, be the reason we observe such large morphological differences in the evoked nonlateralized waveforms. There were only 6 possible Landolt C orientations and the stimuli shared many low-level features. In contrast, our real world objects never repeated and shared few low-level features. This likely leads to a proactive interference such that:

(1) The N2 is not modulated because the first instance of a new target is not sufficiently novel from prior experiences with similar targets (as in the Landolt C condition in the current study) or same targets (Courchesne et al., 1975).
(2) Proactive interference from previous experience with the target decreases the rate of recognition when a target repeats, which may explain why we did not observe an increase in accuracy with repeated presentations of a Landolt C target.

In regard to the role of semantic information in enhancing processing for repeated targets, it is notable that we did not replicate previously reported modulation of the P170 component with target repetition (Woodman et al., 2013). The previously observed modulation of the P170 was thought to reflect increasing links between a given target and LTM representations, consistent with prior work involving stimuli that were difficult to verbalize (Voss et al., 2010). Importantly, although one might expect that our Real World Stimuli would have stronger semantic associations, leading to strong ties to LTM and therefore clear modulation of the P170 component, prior work has indicated that stimuli that are easy to verbalize may be represented in a fundamentally different manner (Danker et al., 2008). Therefore, perhaps nameable stimuli like those used in the current study link to LTM in a fundamentally different manner than more difficult to verbalize Landolt Cs. Consistent with this idea, we found large differences during the N400 time window, which is associated with semantic processing or perceptual fluency. The striking absence of an N400 component with Landolt Cs in Experiment 1 highlights the possibility that using simple laboratory stimuli that repeat throughout an experiment may discourage the semantic processing associated with more realistic stimuli.

We based on our initial analysis plan for nonlateralized components on prior research that had found that the P170, P3b, and LPC were modulated by target repetition (Gunseli et al., 2014). In contrast, it appears that real world stimuli evoke a very different response where the anterior N2, N400 and NSW may be more appropriate components to evaluate. We argue that the more negative-going morphology and associated components may reflect processing that is closer to how repeated targets are processed in the real world, but much more work needs to be done to evaluate this claim. Based on these results, we believe that researchers interested in electrophysiological correlates of repeated targets should expand their palette of potential components of interest depending on the stimuli employed.

### Contralateral Delay Activity

Despite large differences in nonlateralized activity, we found that the decrease in CDA amplitude with repetition was consistent across simple laboratory stimuli and real world objects. This suggests that working memory plays a similar role in representing the target template for both types of stimuli, despite apparent differences in the degree to which semantic processing was engaged. One prediction based on this pattern of results is that if working memory plays a lesser role as a target repeats, correlations with search performance should decrease as the target repeats. Our group recently tested this hypothesis and, across six experiments, found no evidence in favor of it. The correlation between VWM capacity and search performance was equivalent for novel targets and repeated targets that were ostensibly represented in LTM (Williams and Drew, 2018). Of course, all of this evidence is correlational rather than causal. One exception to this rule is recent work with rTDCS from Reinhart and Woodman (2014). They found that, rather than affecting CDA amplitude, rTDCS to the medial-frontal cortex led to enhancement of signals associated with LTM (the P170). Thus, there is converging evidence that there is still much to learn about the respective roles that LTM and VWM representations play in allowing humans to find targets in search arrays.

### Future Directions

In the current work, we observed an unexpectedly large difference in the activity evoked when preparing to search for artificial or real world stimuli. As a result, rather than focusing on 1 or 2 ERP components, we examined a large number of components in an effort to broadly characterize the observed differences. Another approach would be to use time-frequency analyses to try to assess how processing differs for artificial versus real world search targets. Without source localization via concurrent fMRI or high-density recordings, deconvolving the responses for overlapping ERP responses is notoriously difficult (e.g., Verleger, 1997). Some prior work has compared N2pc, CDA and event related alpha synchronization in a subitizing task (Pagano et al., 2015). They found that alpha desynchronization played a similar role for the both the N2pc and the CDA. In future work, we hope to determine whether this effect replicates with real world and artificial stimuli, and to extend these analyses to the suite of nonlateralized components analyzed in the current study.

## Conclusion

When targets repeat, working memory representation of the target prior to the search array decreases irrespective of whether the target is a simple laboratory stimulus like a Landolt C or a picture of a Real World Object. Despite this similarity, we observed large differences in how our subjects processed these two categories of stimuli. These differences suggest that Real World Objects more readily engage LTM resources, resulting in larger behavioral benefits associated with target repetition and large differences in the evoked nonlateralized electrophysiological response. Our results clearly illustrate that stimulus category fundamentally alters the processing of potential targets as we prepare to search for these targets.

It is beyond the scope of the current investigation to determine the underlying causes of the observed differences in processing associated with real world objects or Landolt Cs. We have identified a number of likely candidates, but further research will be needed to determine whether the observed differences are caused by the fact that real world objects are associated with increased semantic meaning, stronger LTM traces due to experience, more readily verbalized, or simply more visually complex. The current work is the first step in identifying the underlying mechanisms that dictate the large changes in processing associated with different stimulus types and lays a rich foundation for future research.

## Ethics Statement

This study was carried out in accordance with the recommendations of the University of Utah Institutional Review Board with written informed consent from all subjects. All subjects gave written informed consent in accordance with the Declaration of Helsinki. The protocol was approved by the University of Utah Institutional Review Board.

## Author Contributions

CJ and LW conducted the research. TD, CJ, and LW designed the research and analyzed the data. TD, CJ, LW, and RL wrote and edited the paper.

## Conflict of Interest Statement

The authors declare that the research was conducted in the absence of any commercial or financial relationships that could be construed as a potential conflict of interest.
