# Erratum: Neural Processing of Repeated Search Targets Depends Upon the Stimuli: Real World Stimuli Engage Semantic Processing and Recognition Memory

**DOI:** 10.3389/fnhum.2018.00516

**Published:** 2018-12-17

**Authors:** Frontiers Production Office

**Affiliations:** Frontiers Media SA, Lausanne, Switzerland

**Keywords:** visual working memory, visual search, ERPs, CDA, N2pc, anterior N2, N400, LPC

Due to a production error, affiliation 2 and 3 were inverted and therefore attributed to the wrong authors.

The correct affiliation for Christopher Michael Jones is: Department of Psychology, The Ohio State University, Columbus, OH, United States. The correct affiliation for Roy Luria is: Sagol School of Neuroscience and the School of Psychological Science, Tel Aviv University, Tel Aviv, Israel.

The publisher apologizes for this mistake. The original article has been updated.

